# Stimulating the Right Temporoparietal Junction with tDCS Decreases Deception in Moral Hypocrisy and Unfairness

**DOI:** 10.3389/fpsyg.2017.02033

**Published:** 2017-11-23

**Authors:** Honghong Tang, Peixia Ye, Shun Wang, Ruida Zhu, Song Su, Luqiong Tong, Chao Liu

**Affiliations:** ^1^Business School, Beijing Normal University, Beijing, China; ^2^State Key Laboratory of Cognitive Neuroscience and Learning and IDG/McGovern Institute for Brain Research, Beijing Normal University, Beijing, China; ^3^Center for Collaboration and Innovation in Brain and Learning Sciences, Beijing Normal University, Beijing, China; ^4^Beijing Key Laboratory of Brain Imaging and Connectomics, Beijing Normal University, Beijing, China

**Keywords:** deception, fairness, moral hypocrisy, impression management, self-deception, transcranial direct current stimulation (tDCS), right temporoparietal junction (rTPJ)

## Abstract

Self-centered and other-regarding concerns play important roles in decisions of deception. To investigate how these two motivations affect deception in fairness related moral hypocrisy, we modulated the brain activity in the right temporoparietal junction (rTPJ), the key region for decision making involved in self-centered and other-regarding concerns. After receiving brain stimulation with transcranial direct current stimulation (tDCS), participants finished a modified dictator game. In the game, they played as proposers to make allocations between themselves and recipients and had a chance to deceive by misreporting their totals for allocations. Results show that deception in moral hypocrisy was decreased after anodal stimulation than sham and cathodal stimulation, only when participants know that their reported totals (appearing fair) would be revealed to recipients rather than being unrevealed. Anodal stimulation also increased offers to recipients than cathodal stimulation regardless of the revelation of reported totals. These findings suggest that enhancing the activity of rTPJ decreased deception caused by impression management rather than self-deception in moral hypocrisy and unfairness through facilitating other-regarding concerns and weakening non-material self-centered motivations. They provide causal evidence for the role of rTPJ in both other-regarding concerns and non-material self-centered motivations, shedding light on the way to decrease moral hypocrisy.

## Introduction

Deception is commonly used in social interaction, in which liars often intentionally and strategically give false statements to mislead others. Motivations that affect deception have attracted researchers’ attention for years. Although people lie mostly for material benefits for themselves, they also lie for non-material self-centered factors, such as regulating feelings or improving self-presentation (DePaulo et al., 1996; Toma et al., 2008). Those non-material self-centered motivations in deception, which aim at making people appear kinder, fairer, smarter or more attractive instead of being truly so, are consistent with motivations in moral hypocrisy that has been commonly defined as the phenomenon to appear moral instead of being truly moral (Batson et al., 1997, 1999).

Moral hypocrisy is closely linked with deliberate or unconscious deception. It has been proposed to be caused by impression management which aims to protect one’s social image in other’s eyes through deception and self-deception that targets on protecting one’s self-concept of morality when people transgress moral principles (Batson et al., 1999, 2002; Valdesolo and DeSteno, 2008). These non-material self-centered motivations make moral hypocrisy sensitive to both social contexts and threats of moral self. Considering the different directions of them, they might lead people to behave differently when appearing moral would be perceived by others than not.

Moral hypocrisy could be classified into different forms based on the existence of public claims (Graham et al., 2015). Moral deception or moral duplicity that observed when people appear fair through flipping a coin but misreporting the results of the coin (Batson et al., 1999, 2002; Lönnqvist et al., 2014) and moral double standards that used in moral judgment when people evaluate their own moral transgressions less harshly than others (Valdesolo and DeSteno, 2007, 2008) have been treated as interpersonal moral hypocrisy. Moral weakness which describes the conflicts between moral values and behaviors, can exist without public claims, is classified as intrapersonal moral hypocrisy. Although interpersonal moral hypocrisy could engage self-deception to make it more successful through dealing threat of moral self (Batson et al., 1999), it essentially relies on social context and might be more sensitive to changes driven by impression management than self-deception.

Researchers also try to reduce moral hypocrisy and most of them focus on changing the processing of self-concept. For example, some of them found that increasing concerns of self-concept can reduce moral hypocrisy by increasing the self-awareness with a mirror (Batson et al., 1999) or priming religious motivations through religious concepts (Carpenter and Marshall, 2009). Others show that increasing cognitive load to limit cognitive processing of protecting self-concept can also decrease moral hypocrisy (Valdesolo and DeSteno, 2008). However, how concerns of others affect interpersonal moral hypocrisy is still ambiguous. Studies have found that people show other-regarding concerns when they decide whether to deceive or not. They care about the harms, losses or feelings of others in deception (Biziou-van-Pol et al., 2015). Half of honest people are led by other-regarding preferences to be honest (Sheremeta and Shields, 2013), and people decrease deception and lower perceived fairness of deception when they consider the loss of others (Gneezy, 2005). Another study also shows that imaging others’ thoughts and feelings in the same situation reduce moral hypocrisy (Batson et al., 2003), indicating the role of other-regarding concerns in moral hypocrisy. However, other-regarding concerns might either decrease interpersonal moral hypocrisy through leading people to be actually prosocial and care others’ feelings and payoffs, or increase interpersonal moral hypocrisy by enhancing the self-centered motivation to endorse other-regarding moral principles to protect ones’ social image (Szabados and Soifer, 2004). Although both these two accesses require the perspective-taking mechanism, they have opposite effects on interpersonal moral hypocrisy. Thus, in the current study, we modulated the other-regarding concerns through brain stimulation techniques to investigate how it would affect moral hypocrisy.

Neural imaging studies show that the right temporoparietal junction (rTPJ) is a key brain region for social cognition and decision making involved in self and other presentations (Decety and Lamm, 2007; Murray et al., 2012). On the one hand, activity in rTPJ is engaged in understanding other’s mental states in theory of mind (Saxe et al., 2006; Van der Meer et al., 2011). It contributes to successful strategic deception in social interaction through inferring other’s beliefs and intentions (Bhatt et al., 2010; Tang et al., 2015). On the other hand, rTPJ is active in decisions involved self-centered and other-regarding concerns. When facing the choices between selfish and generous alternatives, TPJ inhibits selfish motivation then facilitates generosity (Strombach et al., 2015). The activity in rTPJ is also associated with altruistic allocations in dictator game (Morishima et al., 2012), and altruistic third-party punishment for unfair behaviors (David et al., 2017).

Recent studies also show causal links between the function of the rTPJ and self-centered and other-regarding concerns in behaviors with non-invasive brain stimulation techniques. For example, increasing excitability of rTPJ with anodal stimulation of transcranial direct current stimulation (tDCS) enhances performances in perspective-taking task (Santiesteban et al., 2012); decreasing excitability of rTPJ with cathodal stimulation of tDCS weakens cognitive empathy in theory of mind (Mai et al., 2016). Moreover, strengthening TPJ with tDCS increases inequality aversion in advantageous situations (Luo et al., 2017), and disrupting rTPJ with disruptive transcranial magnetic stimulation (TMS) decreases the ability to overcome egocentricity, suppressing pro-social choices (Soutschek et al., 2016). These results indicate that modulating the activity in rTPJ could change both self-centered and other-regarding concerns in behaviors.

In this study, we stimulated the rTPJ with tDCS techniques to explore how non-material self-centered motivations and other-regarding concerns affect fairness related moral hypocrisy. We used a revised version of dictator game, in which participants played as the proposer and had a chance to deceive about the total amount of money units (MUs) for allocation, then made a division between self and the recipient. The recipient cannot reject the allocation, providing the opportunity for participants to act unfairly instead of being unfair through appearing fair and excluding the effects of materialistic self-interest on moral hypocrisy. To investigate the tDCS effect on impression management and self-deception in moral hypocrisy, we manipulated whether participants’ reported totals of allocation would be revealed to recipients or not. We predicted participants to deceive more when the reported totals would be revealed than unrevealed for they concern social image. And this discrepancy would be changed by increasing other-regarding concerns through tDCS stimulation on rTPJ.

## Materials and Methods

### Participants

Ninety-six participants [58 females, age (mean ± SD): 22.36 ± 2.37] were recruited as proposers in two waves (72 participants in the first wave and 24 participants in the second wave). They were randomly assigned into the anodal [*n* = 32 (7 in the second wave)), cathodal (*n* = 30 (5 in the second wave)] or sham group [*n* = 34 (12 in the second wave)]. One participant in the sham group who was skeptic about the tDCS stimulation in the first wave, and three participants in the second wave who said that they thought the recipients were not real humans (one in the anodal group and two in the sham group) was excluded in the analysis (final *N* = 92). All participants were healthy students and paid according to their performances in the experiment (about 40–50 RMB). This study was approved by the Institutional Review Board of the State Key Laboratory of Cognitive Neuroscience and Learning at Beijing Normal University.

### Procedure and Design

A 3 (tDCS: Anodal vs. Cathodal vs. Sham) × 2 (Revelation: Reported Revealed vs. Unrevealed) mixed design was run, in which the tDCS was a between-subject factor and the revelation of reported totals (whether the recipient would know the reported totals) was a within-subject factor. Firstly, participants filled the Interpersonal Reactivity Index scale (IRI) (Davis, 1980) which measures the tendency of empathy and then randomly received either anodal, cathodal or sham stimulation over the rTPJ with a constant-current stimulator (DC-Stimulator Plus, NeuroConn GmbH, Germany). A saline-soaked pair of surface sponge electrodes (in 35 cm^2^ size) was used, in which the anodal or cathodal one was placed over P6 and CP6 in the international 10–20 EEG system in the brain (Jurcak et al., 2007; Santiesteban et al., 2012), and the reference one was placed over the left cheek. With a current of 1.5 mA, 15 s fade in and fade out, participants in the anodal and cathodal groups received 20 min stimulation, and participants in the sham group received anodal stimulation for 15 s (Keeser et al., 2011; Santiesteban et al., 2012).

**FIGURE 1:**
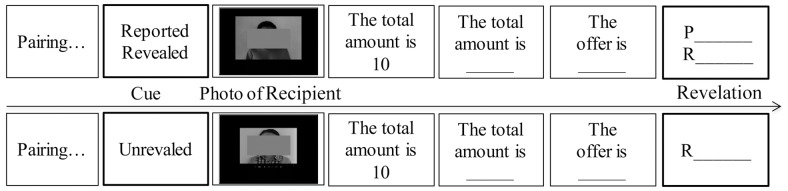
The procedure of the experiment with two example trials that proposers were told that both gains of P (proposer) and R (recipient) would be revealed (Reported Revealed) or only the offer to R would be revealed (Unrevealed).

Next, all participants were instructed to play a dictator game (Forsythe et al., 1994), in which participants played as the proposer and made a division between themselves and different recipients in 32 trials (photos of confederate recipients were shown). Participants were instructed to play with different real recipients whose photos were collected before the experiment and would be shown in each trial. They were told that they would randomly gain a total amount of money for allocation from the computer and the amount would be only known by themselves (four monetary units [MUs] (8, 10, 12, or 14) were randomly extracted in each trial and the range was not told to participants). Next, they needed to report an amount of the money for allocation (providing a chance to tell a lie) and made a division between themselves and recipients. In half of the trails, both their reported totals and offers would be revealed to recipients (Reported Revealed); in another half of trials only offers and nothing about the totals would be revealed to recipients (Unrevealed). Their divisions would determine the payoffs between themselves and the recipients, and recipients would not know the true totals in both conditions. After the instructions, participants answered checking questions including “Will your divisions affect recipients’ payoffs?” “Will your true totals for allocation would be known by others?” “What will the recipient would know in the reported revealed and unrevealed condition?” and practiced to ensure that they understand the game. In each trial, participants would see a screen about pairing recipients for them, then know whether their reported totals would be revealed not before they saw the photos of a recipient. After that, they gained the total for allocation, reported the total and made the offer to the recipient. Finally, the gains would be revealed, in which participants were told that recipients would see both gains of them and the offers based on their reported totals in the reported revealed condition or recipients would only see the offers in the unrevealed condition (**Figure 1**).

After they finished the game, they rated how much different they perceived between the reported revealed and unrevealed conditions when they made decisions from 1 (Not different at all) to 7 (Strongly different). They also rated how fair of being the proposer in this game, and how fair the offers 5:5, 7:3, 8:2, 9:1 were from 1 (Not fair at all) to 7 (Strongly fair). Note that the offer 9:1 in this question means when the true total was 10, the proposer kept 9 and offered 1 to the recipient. Then they filled the Positive and Negative Affect Schedule scale (PANAS) (Watson et al., 1988) to measure their emotional states in the experiment. Finally, all of them were debriefed with questions including “What the purpose of this experiment in your opinion?” “How will these recipients feel after the experiment?” They were told the objective of this experiment and were required not to talk this study with others. To check whether they really believed that they played against real humans, participants in the second wave were also required to write down their strategies in the reported revealed and unrevealed conditions, their thoughts about the recipients and who they thought the recipients were. After that, they were also directly asked about whether they regarded recipients as real humans and knew that their divisions would take effect on recipients when they made decisions in the experiment. Only 3 in 24 participants (12.5%) reported that they didn’t believe these recipients were real humans and didn’t consider the recipients’ payoffs would be affected by their divisions. Their data has been excluded in the analysis.

We compared the percentage of participants who actually deceived in each group, analyzed the deception rate [percentage of deceptive trials to all trials (%)], mean magnitude of dishonesty (the true total minus the reported total), and offer proportion (proportion of offers to the true total amount) with 3 (tDCS: Anodal vs. Cathodal vs. Sham) × 2 (Revelation: Reported Revealed vs. Unrevealed) mixed ANOVA.

## Results

**FIGURE 2:**
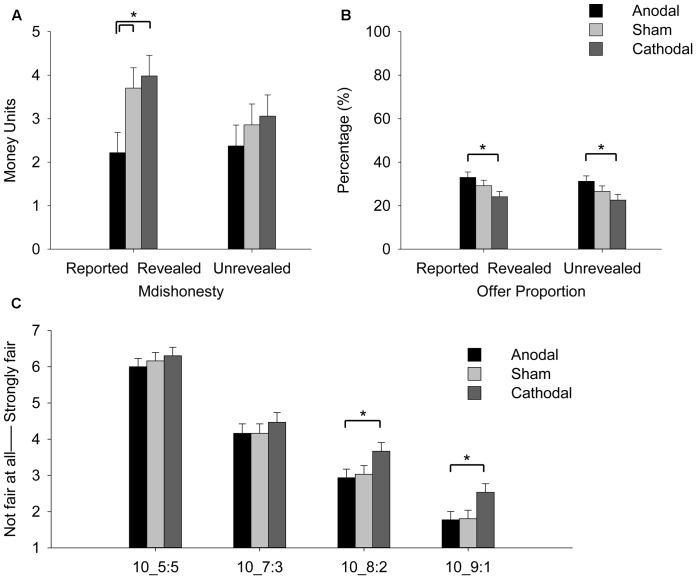
**(A)** Mean magnitude of dishonesty after receiving anodal, cathodal and sham stimulation with tDCS over rTPJ. **(B)** Mean offer proportion after tDCS stimulation. **(C)** Fairness rating of 5:5, 7:3, 8:2, and 9:1 offers based on the true total as 10 after the task (^∗^*p* < 0.05). Error bars indicate standard errors.

Percentage of participants who actually showed deception after receiving anodal stimulation (74%) was less than cathodal (97%) and sham (94%) stimulation in the reported revealed condition [χ^2^(2)= 8.66, *p* = 0.01]. No such difference was found in the unrevealed condition [anodal: 77%, cathodal: 87%, sham: 94%, χ^2^(2)= 3.35, *p* = 0.19]. On the deception rate, main effect of Revelation was found [*F*(1,89) = 12.51, *p* = 0.001, $\eta_{p}^{2}$= 0.12], and no main effect of tDCS or interaction of tDCS × Revelation was significant (*F*s < 2.05, *ps* > 0.14) (**Table 1**). Analysis on the magnitude of dishonesty showed significant main effect of Revelation [*F*(1,89) = 8.05, *p* = 0.006, $\eta_{p}^{2}$= 0.08] and significant interaction of tDCS × Revelation [*F*(2,89) = 3.37, *p* = 0.039, $\eta_{p}^{2}$= 0.07] (**Figure 2A**). Participants had greater dishonesty in the reported revealed condition than in the unrevealed condition only in the cathodal [*t*(29) = 2.17, *p* = 0.038] and sham [*t*(30) = 2.53, *p* = 0.02] groups but not in the anodal group. The tDCS effect was significant in the reported revealed [*F*(2,89) = 4.07, *p* = 0.02, $\eta_{p}^{2}$= 0.08] but not in the unrevealed condition [*F*(2,89) = 0.54, *p* = 0.58]. That is, anodal stimulation on rTPJ reduced dishonesty than cathodal [*t*(59) = -2.68, *p* = 0.01, Cohen’s *d* = 0.68] and sham [*t*(60) = -2.19, *p* = 0.03, Cohen’s *d* = 0.60] stimulation in the reported revealed but not in the unrevealed condition (*t*s < 0.97, *ps* > 0.34).

**Table 1:** Deception rate (%), response time (RT: ms) when participants reported the total and made the offer (mean).

	Deception rate (%)		Reporting totals (RT: ms)		Making offers (RT: ms)	
	Reported Revealed	Unrevealed	Reported Revealed	Unrevealed	Reported Revealed	Unrevealed
Anodal	54	52	1265	1382	1198	1211
Sham	71	57	1166	1293	1168	1209
Cathodal	70	57	1273	1276	1087	1223


Analysis on offer proportion showed significant main effect of Revelation [*F*(1,89) = 15.50, *p* < 0.001, $\eta_{p}^{2}$= 0.15] and marginally significant main effects of tDCS [*F*(1,89) = 3.06, *p* = 0.052, $\eta_{p}^{2}$= 0.06] (**Figure 2B**). No significant interaction of tDCS × Revelation [*F*(2,89) = 0.38, *p* = 0.69, $\eta_{p}^{2}$= 0.008] was found. Anodal stimulation significantly increased their offers than cathodal stimulation in both reported revealed [*t*(59) = 2.26, *p* = 0.02, Cohen’s *d* = 0.60] and unrevealed conditions [*t*(59) = 2.23, *p* = 0.03, Cohen’s *d* = 0.57]. The main effect of tDCS on fairness was also marginally significant in the rating of four offers (5:5, 7:3, 8:2 and 9:1) [*F*(1,89) = 2.47, *p* = 0.09, $\eta_{p}^{2}$= 0.05], in which anodal stimulation significantly and sham stimulation marginally decreased the fair ratings of the offers 8:2 [anodal: *t*(59) = -2.01, *p* = 0.049, Cohen’s *d* = 0.51; sham: *t*(59) = -1.77, *p* = 0.08, Cohen’s *d* = 0.46] and 9:1 [anodal: *t*(59) = -2.12, *p* = 0.038, Cohen’s *d* = 0.54; sham: *t*(59) = -2.00, *p* = 0.05, Cohen’s *d* = 0.51] (**Figure 2C**).

No significant difference was found for the response time either when participants reported the total or when participants made the offer (see response time in two conditions in **Table 1**) (*Fs* < 2.07, *ps* > 0.13), indicating that our results were not caused by tDCS changed participants’ cognitive ability in this game. Participants’ perceived difference between the two conditions in decisions (anodal: 3.48 ± 2.06; sham: 3.58 ± 1.98; cathodal: 3.17 ± 1.97) and perceived fairness of being the proposer in this game (anodal: 3.35 ± 1.70; sham: 2.81 ± 1.49; cathodal: 3.50 ± 1.96) were not affected by tDCS stimulation on rTPJ (*Fs* < 1.38, *ps* > 0.26). In addition, IRI scores (including perspective taking, fantasy, and empathic concern) before the brain stimulation (*Fs* < 1.76, *p*s > 0.18), and PANAS scores at the end of the experiment were not different among three groups (*Fs* < 2.58, *p*s > 0.08). These results excluded the possibilities that difference of participants’ behaviors was caused by their essential perception of the conditions or being the proposers *per se*, or they were different in empathy or emotional state.

## Discussion

The present study examined the role of self-centered and other-regarding concerns in deception in fairness related moral hypocrisy through stimulating rTPJ by tDCS. We found that deception in moral hypocrisy was increased by revealing appearing fair without true fairness to recipients than not and this effect was decreased by anodal stimulation on rTPJ rather than cathodal and sham stimulation. Anodal stimulation on rTPJ increased truly fairness than cathodal stimulation regardless of the revelation of appearing fair and led participant to rate extremely unfair offers less fair. These findings suggest that exciting activity in rTPJ increases other-regarding concerns then increases truly fair behaviors. Specifically, it decreases non-material self-centered deception in moral hypocrisy when social image concerns exist but not when social image concerns are lacking.

Previous studies have discussed how rTPJ contributes to deception through understanding other’s minds (Bhatt et al., 2010; Tang et al., 2015). In those cases, rTPJ processes beliefs or intentions of others, and helps to build one’s reputation in social interaction then assists deception. However, our findings confirmed the causal role of rTPJ in deception with a different access. In the current study, it is unnecessary for participants to mentalize how recipients’ responses would affect their own gains in the current trial, or to build the reputation for future materialistic reward. The repeated one-shot dictator game in which the recipients cannot reject allocations and recipients were different in each trial removed effects of both current and long-term social interaction and material reward on deception.

Results that enhancing rTPJ decreased the deception in moral hypocrisy provided more information for this access. When the reported total would be revealed, it is hard to separate the effects of self-deception and impression management motivations in moral hypocrisy. In contrast, when the reported total is not revealed, it lacks social image concerns then leads self-deception motivation to be more prominent (Batson et al., 2002; von Hippel and Trivers, 2011). Our findings that participants deceived a lot in the unrevealed condition confirmed the existing of self-deception in moral hypocrisy, and the cathodal and sham group deceived more in the reported revealed than in the unrevealed condition support that participants concerned social image in other’s eyes. Moreover, anodal stimulation decreased the difference of deception between these two conditions through decreasing deception in the reported revealed condition, suggesting that rTPJ is only involved in moral hypocrisy driven by impression management but not by self-deception.

Exciting rTPJ increased truly fair behaviors provided further explanation for these results. In line with previous findings that rTPJ inhibits selfish motivations to maximize materialistic benefit and facilities other-regarding behaviors in allocation (Morishima et al., 2012; Strombach et al., 2015; Luo et al., 2017), our results show that exciting rTPJ increased other-regarding concerns regardless of whether fairness would be perceived or not. Moreover, the enhancement of other-regarding concerns decreases deception in moral hypocrisy driven by concerns of social image rather than increasing the moral hypocrisy by endorsing other-regarding moral principles to protect one’s social image (Szabados and Soifer, 2004). These findings provide causal evidence for the role of other-regarding concerns in reducing moral hypocrisy (Batson et al., 2003; Graham et al., 2015), and indicate that this effect might be caused by TPJ constructs social contexts through integrating social information and reorients people’s attention to social stimuli (Carter and Huettel, 2013), then exciting rTPJ prompts people to pay more attention to interpersonal processes involved in impression management (Schlenker and Weigold, 1992). That is, increasing other-regarding concerns facilitates considering other’s evaluations and expectations, therefore, decreases deception in moral hypocrisy driven by impression management rather than self-deception.

Another possibility is that rTPJ is not involved in self-deception processing. Recently, researchers investigated the neural correlates of self-deception and impression management with the Balanced Inventory of Desirable Responding (BIDR) scale through functional magnetic resonance imaging (fMRI) technique (Farrow et al., 2015; Paulhus, unpublished). They found that impression management is correlated with activity in the left TPJ, whereas self-deception is not correlated with activity in bilateral TPJ. As the authors noted, the reason why the fMRI study did not find the relationship between impression management and rTPJ might be they did not directly measure participants’ hypocritical behaviors based on impression management and self-deception (Farrow et al., 2015). In line with this study, we found that the rTPJ is engaged in processing one’s public image but not in promoting self-concept. One potential explanation is self-deception involves the mechanisms for action selection and interpretation to justify self-serving unethical behaviors and diminish the threat to moral self (Mijović-Prelec and Prelec, 2010; Shaul et al., 2015). These mechanisms might be more closely related to cognitive control system since increasing cognitive load disrupts rationalization and justification of one’s own moral transgressions (Valdesolo and DeSteno, 2008) and repeatedly exposing the truth decreases self-deception (Chance et al., 2015).

One limitation of this study is it is hard to obtain the baseline of self-deception in the interpersonal moral hypocrisy which essentially involves concerns of both self and others. Future studies using intrapersonal moral hypocrisy paradigms would provide more evidence for how self-centered and other-regarding concerns affect self-deception. Another limitation is we used photos of real humans to construct the social context between participants and recipients rather real participants. However, only very few participants suspected that they played against real recipients, indicating that most participants decreased moral hypocrisy for protecting the social image.

Taken together, our study is the first one to investigate how activity in rTPJ affects deception in fairness related moral hypocrisy. The results support that rTPJ is involved in other-regarding behaviors and contributes to decreasing deception in moral hypocrisy through facilitating interpersonal processes. Future studies about how cognition for deception and fairness is processed in moral hypocrisy would be helpful to understand the role of rTPJ in decisions for non-material reward.

## Ethics Statement

This study was carried out in accordance with the recommendations of Institutional Review Board of the State Key Laboratory of Cognitive Neuroscience and Learning at Beijing Normal University with written informed consent from all subjects. All subjects gave written informed consent. The protocol was approved by the Institutional Review Board of the State Key Laboratory of Cognitive Neuroscience and Learning at Beijing Normal University.

## Author Contributions

HT, SW, and CL designed experiment. HT, PY, SW, and LT performed experiment. RZ and LT analyzed data and drew figures. HT, PY, SW, RZ, SS, and CL wrote the manuscript. HT, RZ, SS, LT, and CL revised the manuscript and HT, PY, SW, RZ, SS, LT, and CL finally approved the version to be published.

## Conflict of Interest Statement

The authors declare that the research was conducted in the absence of any commercial or financial relationships that could be construed as a potential conflict of interest.
