# Corrigendum: Stimulating the Right Temporoparietal Junction with tDCS Decreases Deception in Moral Hypocrisy and Unfairness

**DOI:** 10.3389/fpsyg.2018.00142

**Published:** 2018-02-09

**Authors:** Honghong Tang, Peixia Ye, Shun Wang, Ruida Zhu, Song Su, Luqiong Tong, Chao Liu

**Affiliations:** ^1^Business School, Beijing Normal University, Beijing, China; ^2^State Key Laboratory of Cognitive Neuroscience and Learning and IDG/McGovern Institute for Brain Research, Beijing Normal University, Beijing, China; ^3^Center for Collaboration and Innovation in Brain and Learning Sciences, Beijing Normal University, Beijing, China; ^4^Beijing Key Laboratory of Brain Imaging and Connectomics, Beijing Normal University, Beijing, China

**Keywords:** deception, fairness, moral hypocrisy, impression management, self-deception, transcranial direct current stimulation (tDCS), right temporoparietal junction (rTPJ)

In the original article, the name of one of the authors was incorrectly spelled in the reference for Mijović-Prelec, D., and Prelec, D. (2010). Self-deception as self-signaling: a model and experimental evidence. *Philos. Trans. R. Soc. B* 365, 227–240. doi: 10.1098/rstb.2009.0218 as Prelec (2010). It should be Mijović-Prelec and Prelec (2010). The authors apologize for this error and state that this does not change the scientific conclusions of the article in any way.

The original article has been updated.

## Conflict of interest statement

The authors declare that the research was conducted in the absence of any commercial or financial relationships that could be construed as a potential conflict of interest.
